# Dietary patterns in pregnancy and birth weight

**DOI:** 10.1590/S0034-8910.2015049005403

**Published:** 2015-09-29

**Authors:** Natália de Lima Pereira Coelho, Diana Barbosa Cunha, Ana Paula Pereira Esteves, Elisa Maria de Aquino Lacerda, Mariza Miranda Theme Filha

**Affiliations:** I Programa de Pós-Graduação de Epidemiologia em Saúde Pública. Escola Nacional de Saúde Pública. Fundação Oswaldo Cruz. Rio de Janeiro, RJ, Brasil; II Departamento de Epidemiologia. Instituto de Medicina Social. Universidade do Estado do Rio de Janeiro. Rio de Janeiro, RJ, Brasil; III Instituto de Nutrição Josué de Castro. Universidade Federal do Rio de Janeiro. Rio de Janeiro, RJ, Brasil

**Keywords:** Pregnant Women, Prenatal Nutrition, Pregnancy Third Trimester, Food Consumption, Birth Weight

## Abstract

**OBJECTIVE:**

To analyze if dietary patterns during the third gestational trimester are associated with birth weight.

**METHODS:**

Longitudinal study conducted in the cities of Petropolis and Queimados, Rio de Janeiro (RJ), Southeastern Brazil, between 2007 and 2008. We analyzed data from the first and second follow-up wave of a prospective cohort. Food consumption of 1,298 pregnant women was assessed using a semi-quantitative questionnaire about food frequency. Dietary patterns were obtained by exploratory factor analysis, using the Varimax rotation method. We also applied the multivariate linear regression model to estimate the association between food consumption patterns and birth weight.

**RESULTS:**

Four patterns of consumption – which explain 36.4% of the variability – were identified and divided as follows: (1) prudent pattern (milk, yogurt, cheese, fruit and fresh-fruit juice, cracker, and chicken/beef/fish/liver), which explained 14.9% of the consumption; (2) traditional pattern, consisting of beans, rice, vegetables, breads, butter/margarine and sugar, which explained 8.8% of the variation in consumption; (3) Western pattern (potato/cassava/yams, macaroni, flour/*farofa*/grits, pizza/hamburger/deep fried pastries, soft drinks/cool drinks and pork/sausages/egg), which accounts for 6.9% of the variance; and (4) snack pattern (sandwich cookie, salty snacks, chocolate, and chocolate drink mix), which explains 5.7% of the consumption variability. The snack dietary pattern was positively associated with birth weight (β = 56.64; p = 0.04) in pregnant adolescents.

**CONCLUSIONS:**

For pregnant adolescents, the greater the adherence to snack pattern during pregnancy, the greater the baby’s birth weight.

## INTRODUCTION

Birth weight is an important indicator of child survival and reflects the risk of death and hazards to health during the first year of life.^a^ Several factors are related to variations in birth weight, standing out the maternal food consumption.^15^ It is essential for the pregnant diet sufficient energy and nutrients to maintain maternal health, to allow for adequate fetal growth and development.^7^ ^,^ ^30^ However, assessing food consumption becomes a challenge due to the human diet complexity, something that solicits researchers to refine assessment methods of food consumption.

Historically, most epidemiological studies had the main focus on assessing the association between consumption of nutrients and disease development.^20^ However, this evaluation is considered reductionist because it disregards the complex interaction between nutrients and foods. This occurs because individuals do not ingest isolated nutrients but meals composed of a wide variety of foods that interact with each other.^29^

Addressing food consumption with the identification of food patterns has been described as a method that are close to the complex act of feeding,^31^ given that it considers different combinations and compositions of meals. Moreover, the study of food patterns allows the formulation of food-based dietary recommendations.^31^

However, few studies have assessed the pattern of food consumption among pregnant women, particularly its effects on the foetus.^10^ ^,^ ^22^

This study aimed to analyze whether dietary patterns during the third gestational trimester are associated with birth weight.

## METHODS

This study is part of the project *Capital Social e Fatores Psicossociais associados à Prematuridade e ao Baixo Peso ao Nascer* (Social Capital and Psychosocial Factors associated with Prematurity and Low Birth Weight) conducted in the cities of Petropolis and Queimados, RJ, Southeastern Brazil, between December 2007 and August 2008.^b^ It is a prospective cohort with four waves of follow-up: pregnancy, puerperium, three and six months after birth. We analyzed data from the first (gestation) and the second stage (postpartum) of follow up.

All pregnant women with up to 22 weeks of gestation were invited to participate in the cohort, assisted by prenatal services of 11 municipal health units of Unified Health System (SUS) – eight in Queimados and three in Petropolis, RJ, which had 90.0% of the prenatal care coverage of SUS.

For the calculation of the cohort sample size, we considered a prevalence of 10.0% of premature and low weight births, a significance level of 5% and 90.0% of power to detect differences of at least 5.0%.^11^ After adjustments for finite populations and increase of 20.0% for possible losses during the follow-up of pregnant women, the sample size was established with 940 pregnant women in Petropolis and 840 in Queimados.

Due to premature births (n = 167), loss of follow-up (n = 104), refusals (n = 71), miscarriages (n = 62), lack of information on birth weight (n = 31), and occurrence of twin pregnancy (n = 17), the sample of this study was composed of 1,298 women.

Food consumption was assessed with a semi-quantitative food frequency questionnaire (FFQ), referring to the last gestational quarter, with 29 food items and obtained by simplifying a 80-item FFQ, validated by Sichieri and Everhart.^28^ To this simplification, we performed a secondary analysis of the database employed in the elaboration of the original FFQ, using stepwise regression to explain 95.0% of variance for the following nutrients: energy, carbohydrates, lipids, saturated fatty acids, monounsaturated and polyunsaturated fats, cholesterol, calcium and vitamin C.^c^

The birth weight information came from medical records. When data were not available, we analyzed child booklets or certificates of born alive.

The following variables were evaluated: (i) sociodemographic: age of pregnant women, marital status (lives with a partner; does not live with a partner), self-referred skin color (white; black; mixed race), education level according to years of study, work outside home (no; yes), economic class (B or C; D or E); (ii) tobacco (no; yes); (iii) self-referred chronic diseases: arterial hypertension (not; yes), diabetes mellitus (not; yes); (iv) obstetric antecedents: parity (primipara; 2-3 children; 4 or more children), prior history of perinatal death, low birth weight or prematurity; (v) maternal nutritional status (pregestational BMI and gestational weight gain), and (vi) adequacy of prenatal care. The obstetric history and complications in pregnancy, such as diabetes and arterial hypertension, were referred by the woman during the interview in the maternity ward after giving birth with the question: “Did you have any of these health issues during pregnancy?” High blood pressure or eclampsia (yes; no); gestational diabetes (yes; no).

The socioeconomic classification followed the criterion adopted by *Associação Brasileira de Empresas de Pesquisa* (ABEP – Brazilian Association of Research Companies) and was based on the family property and householder’s educational level, which composed the final score that defines socioeconomic groups: A (highest), B, C, D and E (lowest).^1^ Socioeconomic classes were aggregated for multivariate analysis in B or C, and D or E – no women classified in the A category. To assess the adequacy of prenatal care, we used Kotelchuck index,^16^ which is based on the gestational age at the beginning of prenatal care and on the adequacy percentage of the number of visits. These two dimensions were grouped into three categories: prenatal care more than appropriate; appropriate; partly inappropriate; and inappropriate.

The datum about pregestational weight was obtained from the pregnant medical record until the thirteenth gestational or, if unavailable, it was self-reported by the patient. This weight was used to calculate body mass index (BMI) before pregnancy, allowing to classify the nutritional status of pregnant women in four categories: underweight (pregestational BMI < 18.5 kg/m^2^), adequate weight (pregestational BMI between 18.5 and 24.9 kg/m^2^), overweight (pregestational BMI between 25 and 29.9 kg/m^2^) and obesity (pregestational BMI ≥ 30 kg/m^2^).^26^ Based on these information, we determined the ranges of weight adequacy for each expectant mother, which were equivalent to the total gestational weighted gain: 12.5 to 18 kg for women with low pregestational weight; 11.5 to 16 kg for eutrophic women; 7 to 9 kg for overweight women; and 7 kg for obese women.^26^ The weight at the end of pregnancy was referred to by women during the postpartum interview. For the calculation of the total gestational weight gain, we subtracted pregestational weight from weight at the end of pregnancy.

For the identification of dietary patterns, the 29 items listed in FFQ were grouped in 20 food groups based on their nutritional characteristics and consumption frequency.

The exploratory factor analysis was used to derive dietary patterns based on the daily portion of consumption of each food group. A correlation matrix was used to check if the variables were correlated and if the factorial model was appropriate for the data. For this, we used the Bartlett’s sphericity test (BST) and the Kaiser-Meyer-Olkin (KMO) measure of sampling adequacy.

The factor extraction was performed by principal component analysis (PCA), orthogonally transformed, with Varimax rotation to obtain a structure with independent factors, which helped in the interpretation.

The definition of factors to be retained was based on scree plot (a graph that shows eigenvalues in relation to the number of factors in their order of extraction), on the percentage of variance explained by factors and on the knowledge of the researchers (considerations on interpretability and simplicity of patterns). Then a new factor analysis was conducted with the establishment of the number of factors to be extracted.

We kept in the factors (patterns) the items with factor loading higher than 0.30 and considered acceptable minimum commonalities of 0.25.^23^ The internal consistency of each extracted factor was verified by Cronbach’s alpha. Subsequently, the patterns were named based on data interpretation.

The association between dietary patterns and birth weight was assessed by the multivariate linear regression model. Initially, we conducted the bivariate linear regression analysis between all exposure variables and birth weight, in addition to bivariate linear regression analysis between all covariates of the study and dietary patterns to detect possible confounding factors. A separate analysis was conducted for each dietary pattern. All covariates with p < 0.20 in both analyses were considered feasible elements for the final model. The final model was also mutually adjusted by other dietary patterns, given that, in this kind of analysis, individuals participate in more than one pattern, which are not correlated due to the rotation method used.

The study was approved by the Research Ethics Committee of Escola Nacional de Saúde Pública (ENSP/Fiocruz – CAAE 0156.0.031.000-06), in accordance with the ethical principles stipulated by Resolution 196/96 of the National Health Council (CNS, 1996).

The statistical analyses were carried out with the software Statistical Package for Social Sciences (SPSS) for Windows, version 16.0.

## RESULTS

The average age of the women interviewed was 24.7 years, and the average years of study was less than eight. Most women had no paid job (59.7%) and were in the economic classes B or C (66.4%). Most of them already had more than one child and 12.8% had prior history of newborn infants with low birth weight, prematurity or perinatal death (Table 1).

**Table 1:** Sociodemographic characteristics, obstetric antecedents; characteristics of pregnancy, delivery and newborn in cohorts of pregnant women. Petropolis and Queimados, RJ, Southeastern Brazil, 2007-2008.

Variable	Total	
	n	%
Social class		
C + B	862	66.4
E + D	436	33.6
Race/Skin color		
White	440	33.9
Mixed race	557	42.9
Black	301	23.2
Marital status		
Partner	957	73.7
No partner	341	26.3
Paid job		
No	775	59.7
Yes	523	40.3
Prior history of perinatal death, low birth weight or prematurity*		
No	635	87.2
Yes	93	12.8
Parity		
Primipara	570	43.9
2 to 3	558	43.0
4 or more	170	13.1
Pregestational nutritional status		
Low weight	96	8.9
Eutrophic	768	71.2
Overweight	214	19.9
Adequacy of gestational weight gain		
Insufficient	358	32.5
Adequate	330	30.0
Excessive	412	37.5
Adequacy of prenatal care		
More than adequate	508	39.2
Adequate	436	33.7
Inadequate	351	27.1
Gestational hypertensive		
No	1,051	81.0
Yes	246	19.0
Gestational diabetes mellitus		
No	1,266	97.5
Yes	32	2.5
Smoking in pregnancy		
No	1,141	88.0
Yes	155	12.0
Delivery type		
Vaginal	723	55.7
Cesarian section	575	44.3
	Mean	SD
Age (in years)	24.7	6.1
Years of education	7.8	2.9
Birth weight (in grams)	3.25	444.8

* Data do not include primiparas.

Considering the adequacy of number of prenatal visits for gestational age (Kotelchuck index),^16^ more than 70.0% of recent mothers had adequate or more than adequate prenatal care. Of the interviewed women, smoking was reported by 12.0% of them (Table 1); 19.0% were diagnosed with gestational arterial hypertension and 2.5% with diabetes.

It was observed that 28.8% of women started pregnancy under an inadequate nutritional status and, of these, 20.0% were overweight. The average of weight gain in pregnancy was 13.3 kg, but less than 1/3 of women had appropriate weight gain, and 37.5% had excessive gain. There was a predominance of vaginal birth (55.7%) and the average birth weight of newborns was 3,253 g (Table 1).

Initially, we identified seven factors with eigenvalues higher than 1.0, which explained the food consumption variance of 51.3% (data not presented in the tables). Based on the scree plot, eigenvalues and considering interpretability of patterns, four factors should be retained, which, altogether, explained 36.4% of the consumption variability (Table 2).

**Table 2:** Rotated factorial matrix, factor loading, variance and Cronbach’s alpha for the four dietary patterns identified among pregnant women from the city of Petropolis and Queimados, RJ, Southeastern Brazil, 2007-2008.

Food group	Factor loading			
	Prudent	Traditional	Western	Snack
Milk	0.49			
Yogurt	0.62			
Cheese	0.59			
Cracker	0.48			
Poultry/Beef/Fish/Liver	0.42			
Fruit or fresh-fruit juice	0.56			
Beans		0.51		
Rice		0.61		
Sugar		0.64		
Butter/Margarine		0.68		
Bread		0.58		
Vegetables		0.36		
Potato/Cassava/Yam			0.60	
Macaroni			0.69	
Flour/*Farofa*/Grits			0.55	
Soft drinks/Cool drinks			0.45	
Pork/Sausages/Egg			0.44	
Pizza/Burger/Deep fried pastries			0.37	
Chocolate				0.61
Chocolate drink mix				0.47
Sandwich cookie				0.68
Salty snacks				0.65

Eigenvalues	3.28	1.94	1.53	1.26
% of variance explained	14.94	8.80	6.94	5.74
% of accumulated variance explained	14.94	23.74	30.68	36.42
Cronbach’s alpha	0.50	0.52	0.33	0.41

d World Health Organization. The global burden of disease: 2004 update. Geneva; 2008 [cited 2015 Apr 17]. Available from: http://www.who.int/healthinfo/global_burden_disease/2004_report_update/en/

The four patterns of consumption were named and composed as follows: (1) prudent pattern (milk, yogurt, cheese, fruit and natural juice, cracker and chicken/beef/fish/liver), which explained 14.9% of the consumption; (2) traditional pattern (beans, rice, vegetables, bread, butter/margarine, and sugar), which explained 8.8% of the variation in consumption; (3) Western pattern (pizza/hamburger/deep fried pastries, potato/cassava/yams, soft drinks/cool drinks, flour/*farofa*/grits, and pork/sausages/egg, which accounts for 6.9% of the variance; and (4) snack pattern (sandwich cookies, salty snacks, chocolate, and chocolate drink mix), which explains 5.7% of the variance (Table 2).

All food groups were associated to one of the four patterns identified and only one food item was excluded from the analysis (coffee/black tea or mate tea) for having very low commonality and not adjusting in any of the patterns established.

In bivariate analysis, considering the statistical significance lower than 20.0%, the prudent pattern was associated positively with economic class B or C, paid work, age, education level, more than adequate prenatal and excessive weight gain in pregnancy, and was inversely associated with smoking in pregnancy. The pregnant women classified in traditional or Western pattern had some common features, such as: higher parity, smoking in pregnancy and lower pregestational obesity. The pregnant women classified in snack pattern were significantly younger, with less overweight and pregestational obesity, and more inadequate prenatal (Table 3).

**Table 3:** Estimation of bivariate linear regression parameters between dietary pattern and maternal variables in cohort of pregnant women. Petropolis and Queimados, RJ, Southeastern Brazil, 2007-2008.

Variável	Prudent pattern		Traditional pattern		Western pattern		Snack pattern	

	β	p	β	p	β	p	β	p
Social class								
E + D	0.000		0.000		0.000		0.000	
C + B	0.309	< 0.001	-0.089	0.108	-0.171	0.002	0.084	0.177
*Per capita* household income								
≤ 1/2 MW	-0.499	< 0.001	0.047	0.544	0.313	< 0.001	0.074	0.312
> 1/2 and > ≤ 1 MW	-0.156	0.005	0.134	0.095	0.175	0.027	0.181	0.018
> 1 MW	0.000		0.000		0.000		0.000	
Race								
White	0.000		0.000		0.000		0.000	
Mixed race	0.010	0.846	-0.035	0.507	0.049	0.355	-0.011	0.83
Black	-0.126	0.039	-0.007	0.914	0.133	0.031	0.024	0.681
Age (years)	0.012	0.006	-0.006	0.143	-0.004	0.343	-0.047	< 0.001
Education level (years)	0.042	< 0.001	-0.003	0.723	-0.045	< 0.001	0.005	0.574
Marital status								
Partner	0.000		0.000		0.000		0.000	
No partner	-0.143	0.015	-0.008	0.891	0.025	0.676	0.191	0.001
Paid job								
No	0.000		0.000		0.000		0.000	
Yes	0.189	< 0.001	-0.016	0.758	-0.084	0.115	-0.157	0.002
Parity								
Primipara	0.065	0.218	0.031	0.561	-0.080	0.128	-0.263	< 0.001
2 to 3	0.000		0.000		0.000		0.000	
4 or more	-0.199	0.008	0.093	0.218	0.314	< 0.001	0.302	< 0.001
Pregestational nutritional status								
Low weight	-0.005	0.960	0.037	0.713	-0.028	0.774	0.118	0.206
Eutrophic	0.000		0.000		0.000		0.000	
Overweight	-0.123	0.092	-0.047	0.516	-0.062	0.394	-0.207	0.020
Obese	-0.082	0.404	-0.217	0.027	-0.227	0.019	-0.305	0.000
Gestational weight gain (kg)								
Insufficient	-0.041	0.564	-0.026	0.722	0.000	0.996	-0.121	0.071
Adequate	0.000		0.000		0.000		0.000	
Excess	0.094	0.177	0.067	0.342	0.046	0.509	-0.108	0.103
Adequacy of prenatal care								
More than adequate	0.260	< 0.001	0.108	0.043	-0.194	< 0.001	-0.086	0.093
Adequate	0.000		0.000		0.000		0.000	
Inadequate	-0.270	< 0.001	-0.060	0.309	0.161	0.007	0.074	0.192
Gestational hypertension								
No	0.000		0.000		0.000		0.000	
Yes	-0.075	0.250	0.007	0.912	0.043	0.509	-0.073	0.247
Gestational diabetes mellitus								
No	0.000		0.000		0.000		0.000	
Yes	0.026	0.872	-0.547	< 0.001	-0.121	0.464	-0.248	0.116
Prior history of perinatal death, LBW or PMT								
No	0.000		0.000		0.000		0.000	
Yes	-0.029	0.255	-0.051	0.047	0.034	0.184	-0.053	0.038
Smoking								
No	0.000		0.000		0.000		0.000	
Yes	-0.176	0.029	0.226	0.005	0.245	0.002	0.052	0.503
Delivery type								
Vaginal	0.000		0.000		0.000		0.000	
Cesarian section	0.041	0.120	-0.082	0.02	-0.028	0.276	-0.060	0.022

β: linear regression coefficient; LBW: low birth weight; MW: minimum wage; PMT: prematurity

* Dietary pattern as dependent variable and other independent variables.

In relation to birth weight, we observed positive association for the following variables: social class B or C, maternal age, education level, pregestational overweight or obesity, excessive gestational weighted gain, and cesarean delivery. In contrast, not living with a partner, being primipara, starting prenatal care with low weight, having inadequate weight gain, receiving inadequate prenatal care, and smoking during pregnancy were negatively associated with birth weight (Table 4).

**Table 4:** Estimation of bivariate linear regression parameters* between birth weight and maternal variables in cohort of pregnant women. Petropolis and Queimados, RJ, Southeastern Brazil, 2007-2008.

Variable	β	p
Social class		
E + D	0.00	–
C + B	70.93	0.007
*Per capita* household income	0.17	0.005*
Race		
White	0.00	–
Mixed race	-31.61	0.281
Black	-14.44	0.563
Age (years)	5.00	0.014
Education level (years)	9.92	0.021
Marital status		
Partner	0.00	–
No partner	-76.48	0.006
Paid job		
No	0.00	–
Yes	4.22	0.867
Parity		
Primipara	-58.76	0.018
2 to 3	0.00	–
4 or more	-11.34	0.758
Pregestational nutritional status		
Low weight	-46.93	0.318
Eutrophic	0.00	
Overweight	122.40	< 0.001
Obese	221.34	< 0.001
Adequacy of gestational weight gain		
Insufficient	-77.41	< 0.001
Adequate	0.00	
Excess	144.29	0.017
Adequacy of prenatal care		
More than adequate	71.49	0.005
Adequate	0.00	–
Inadequate	-51.64	0.063
Gestational hypertension		
No	0.00	–
Yes	36.08	0.253
Gestational diabetes mellitus		
No	0.00	–
Yes	47.23	0.559
Prior history of perinatal death, LBW or PMT		
No	0.00	–
Yes	-67.52	0.159
Smoking		
No	0.00	–
Yes	-133.71	< 0.001
Delivery type		
Vaginal	0.00	–
Cesarian section	149.76	< 0.001
Baby’s sex		
Male	0.00	–
Female	-111.39	< 0.001

β: linear regression coefficient; LBW: low birth weight; PMT: prematurity

* Birth weight as dependent variable and other independent variables.

The variables that had association with significance level of 20.0% with birth weight and dietary patterns, and which were adjusted in the final model, in addition to other dietary patterns, were: economic class, age, education level of the mother, marital status, parity, pregestational nutritional status, adequacy of prenatal care, perinatal death history, low birth weight or prematurity, smoking during pregnancy and delivery type (Tables 3 and 4).

In the multivariate analysis, we observed positive association between snack pattern and birth weight, after adjustment according to maternal age, education level of the mother, marital status, social class, parity, pregestational BMI, adequacy of prenatal care, previous history of low birth weight/prematurity, smoking during pregnancy, newborn sex and type of delivery (data not shown). This association was observed after the inclusion of maternal age in the model, and not in the crude analysis, which showed the presence of negative confusion. It was observed that the distribution of the average factor score of the snack pattern also differed according to age group (0.44 in women between 10 and 19 years old and -0.13 in women 20 or more years of age) (data not presented in tables). These findings explain the attenuation of the effect observed in the crude analysis. Thus, the final model was stratified by maternal age (10 to 19 years; 20 years or more).

The multivariate linear regression analysis adjusted for confounding factors showed a positive association between the snack pattern and birth weight among pregnant adolescents (Table 5).

**Table 5:** Final multivariate model between birth weight and dietary pattern in cohort of pregnant women. Petropolis and Queimados, RJ, Southeastern Brazil, 2007-2008.

Dietary pattern	Crude model		Adjusted model* and stratified by age			
			10 to 19 years		20 or more	

	β	p	β	p	β	p
Prudent	26.50	0.039	55.35	0.130	12.57	0.455
Traditional	-9.23	0.462	11.45	0.723	19.90	0.241
Western	-15.12	0.214	15.88	0.623	10.17	0.554
Snack	17.76	0.168	56.64	0.040	6.57	0.749

* Multivariate model adjusted for age and maternal education level, marital status, social class, parity, pregestational body mass index, prenatal adequacy, smoking during pregnancy, delivery type, and dietary patterns.

## DISCUSSION

The results of this study show that, in general, pregnant women who had prenatal care at the selected health units (SUS) of Queimados and Petropolis were young and from low socioeconomic class, features similar to other studies with pregnant women and recent mothers who used the services of SUS basic unities.^17^ ^,^ ^24^

Regarding anthropometric assessment of these women, although 64.9% of them were in an adequate nutritional status at the beginning of pregnancy, the prevalence of overweight was high (26.9%). This result is similar to that found in the study of Nucci et al^21^ (2001) who, following up 5,564 pregnant women in six Brazilian cities, found prevalence of 24.7% for overweight and obesity.

The average weight gain in pregnancy (13.3 kg) is similar to that found by Lacerda et al^17^ (2007) among pregnant women living in Rio de Janeiro, in 2001 (13.4 kg). The results for gestational weight gain – 37.4% with excessive gain and 30.0% with adequate weight gain according to BMI – are in line with those found in other studies.^5^ ^,^ ^13^

The information on birth weight show that, in this study, the average weight (3,253 g) was a little bit higher (3,142 g) to the one found in another location.^4^ This average of birth weight can be explained by excluding premature newborns from the sample studied. Thus, the average birth weight found in this study does not reach the range between 3,400 and 3,500 g, expected values of birth weight under optimum conditions of fetal growth.^d^

The factor analysis allowed identifying four dietary patterns that have food in common with dietary patterns identified by other researches.

The prudent pattern, also called “healthy” in other studies with pregnant women, has fruit, fresh-fruit juice, vegetables, fish, and poultry.^7^ ^,^ ^15^ ^,^ ^20^

The traditional pattern – beans, rice, vegetables, bread and butter/margarine – represents foods that are part of the Country’s eating habits and are similar to the traditional pattern found in studies that used the same analysis procedures for the extraction of dietary patterns.^8^ ^,^ ^19^

The Western pattern is composed by foods with high-energy density, low nutritional value (low amount or free from micronutrients) and low financial cost, in addition to containing foods with a high proportion of carbohydrates (macaroni, potato/yam/cassava, flour/*farofa*/grits, soft drinks/cool drinks). This pattern also includes source of proteins such as pork, egg, sausages – the latter with low nutritional quality, as it contains large amounts of saturated fat, cholesterol and sodium. Fast foods (pizza/hamburger/deep fried pastries) are also present in this pattern and have low nutritional value and high energy density.

The snack pattern (sandwich cookies, salty snacks, chocolate, and chocolate drink mix) is also composed of foods of low nutritional value and with high-energy density. In the study conducted by Northstone et al,^20^ we found a dietary pattern called “confectionery” (chocolate, sweets, biscuits, and cakes) that has items in common to the snack pattern.

The variables selected as confusion factors in the final statistical model have a theoretical rationale. Birth weight is an important health indicator and indirectly reflects the living conditions of populations.^4^ Thus, better socioeconomic situations as higher education level of mothers, better social class and greater access to health services during pregnancy are commonly associated with healthy food consumption, as well as with an appropriate birth weight.^4^ ^,^ ^7^ ^,^ ^10^ ^,^ ^20^ The family structure is also an important factor capable of affecting maternal food consumption and birth weight. Women living with a partner and having lower parity tend to have access to a better quality diet and giving birth to babies with higher weight.^6^ ^,^ ^10^ ^,^ ^20^ ^,^ ^27^ Early or advanced maternal age, as well as smoking, have been indicated as risk factors for deviations in birth weight.^6^ At the same time, literature shows that pregnant adolescents, smokers and who consume alcohol during pregnancy have a worse dietary pattern.^4^ ^,^ ^7^ ^,^ ^20^ The type of birth also influences birth weight, because in many cases pregnancy is surgically interrupted before the onset of labor, reducing the time of intrauterine growth and weight gain.^4^ In addition, cesarian section is more frequent among women with gestational intercurrences like diabetes, hypertension, anemia, and other complications.^25^ Many of these diseases area result of the inadequate food consumption of these women.^3^ Finally, the maternal BMI also directly influences maternal food consumption and birth weight.^4^ ^,^ ^9^

The analysis of the relationship between food consumption and birth weight showed that, for pregnant adolescents, the greater the adherence to the snack pattern, the greater the baby’s birth weight. This association was probably observed only among younger pregnant women because they are the biggest consumers of this pattern, since they presented mean positive factor score, whereas this mean was negative among women with 20 years of age or over.

The snack pattern consists of foods with high concentrations of simple carbohydrates, lipids and low amounts of protein and micronutrients. This combination of nutrients gives these foods high-energy density, favoring gestational weight gain.

A diet with a lot of energy is associated with higher gestational weight gain^18^ which, in its turn, is directly related to the baby’s birth weight.^26^ This happens because the higher weight gain during pregnancy increases availability and transfer of amino acids, glucose, free fatty acids and triglycerides from the mother to the foetus by increasing its growth and development.^14^ According to IOM^26^ (2009), for every increase of 1 kg in GPG, there is an increase of 16.7 to 22.6 g in the birth weight.

Uusitalo et al^32^ (2009) assessed the association between dietary patterns and gestational weight gain, identifying seven patterns, of which two were associated to gestational weight gain: fast food and alcohol and fat patterns. The authors concluded that women who belonged to the highest quartile of fast food consumption gained, on average, 1.3 kg more than the lowest quartile of this pattern. On the other hand, women in the highest quartile of alcohol and fat consumption gained on average 0.7 kg less than those from the lowest quartile of consumption of this pattern.

Some methodological limitations of this study should be mentioned. Among them, the small number of foods listed in the FFQ and the lack of standardized portions for all items. In addition, the FFQ used was not tested regarding accuracy of food estimation, but only in relation to nutrients. Despite these limitations, the results obtained by the questionnaire are consistent with other studies, as the one performed by Barros et al,^2^ which used similar questionnaire to assess gestational food consumption of 1,228 adolescents and also found results consistent with the literature.

Another important factor related to subjectivity, which involves factor analysis, is the specificity of results obtained with this technique for the population investigated, making difficult the comparison between studies. Moreover, although most women included in our study live in poverty, the factors related to socioeconomic characteristics and lifestyle had relation to eating habits.

There is consensus in the literature that the evaluation of maternal food consumption during pregnancy is important, and studies that address this subject are still scarce. We have not found studies conducted in Brazil on the effect of maternal dietary pattern on the baby’s birth weight. Thus, this study sought to contribute to determine dietary patterns during pregnancy. The epidemiological importance of these results, based on the cohort study in two Brazilian cities, lies in the provision of subsidies for the rationality of most appropriate nutritional intervention strategies for pregnant women.

## Footnotes

a World Health Organization. The global burden of disease: 2004 update. Geneva; 2008 [cited 2015 Apr 17]. Available from: http://www.who.int/healthinfo/global_burden_disease/2004_report_update/en/

b Projeto: “Capital Social e Fatores Psicossociais Associados a Prematuridade e ao Baixo Peso ao Nascer”. Edital MCT/CNPq/MS-SDTIE-DECIT 26/2006 – Estudo de Determinantes Sociais da Saúde, Saúde da Pessoa com Deficiência, Saúde da População Negra, Saúde da População Masculina. Processo nº 409805/2008-8.

c Consultancy provided by Sichieri R for the development of a simplified questionnaire on dietary pattern in 1998.
