# Supplementary material for: Dietary patterns in pregnancy and birth weight
Source: Rev Saude Publica. 2015 Sep 29;49:62. doi: 10.1590/S0034-8910.2015049005403 (PMC4617437; doi:10.1590/S0034-8910.2015049005403)
Supplement: Translation

# Translation: Padrão de consumo alimentar gestacional e peso ao nascer

**Authors:** Natália de Lima Pereira Coelho, Diana Barbosa Cunha, Ana Paula Pereira Esteves, Elisa Maria de Aquino Lacerda, Mariza Miranda Theme Filha

**Affiliations:** I Programa de Pós-Graduação de Epidemiologia em Saúde Pública. Escola Nacional de Saúde Pública. Fundação Oswaldo Cruz. Rio de Janeiro, RJ, Brasil; II Departamento de Epidemiologia. Instituto de Medicina Social. Universidade do Estado do Rio de Janeiro. Rio de Janeiro, RJ, Brasil; III Instituto de Nutrição Josué de Castro. Universidade Federal do Rio de Janeiro. Rio de Janeiro, RJ, Brasil

**Keywords:** Gestantes, Nutrição Pré-Natal, Terceiro Trimestre da Gravidez, Consumo de Alimentos, Peso ao Nascer

## Abstract

**OBJETIVO:**

Analisar se padrões de consumo alimentar durante o terceiro trimestre gestacional estão associados ao peso ao nascer.

**MÉTODOS:**

Estudo longitudinal realizado nos municípios de Petrópolis e Queimados, RJ, entre 2007 e 2008. Foram analisados dados da primeira e segunda onda de seguimento de uma coorte prospectiva. O consumo alimentar de 1.298 gestantes foi aferido por meio de questionário de frequência alimentar semiquantitativo. Os padrões alimentares foram obtidos por análise fatorial exploratória, utilizando o método de rotação Varimax. Aplicou-se modelo de regressão linear multivariado para estimar a associação entre padrões de consumo alimentar e peso ao nascer.

**RESULTADOS:**

Foram identificados quatro padrões de consumo, que explicam 36,4% da variabilidade, compostos da seguinte forma: (1) padrão prudente, composto por leite, iogurte, queijo, frutas e suco natural, biscoito sem recheio e carne de frango/boi/peixe/fígado, que explica 14,9% do consumo; (2) padrão tradicional, composto por feijão, arroz, vegetais, pães, manteiga/margarina e açúcar, que explica 8,8% da variação do consumo; (3) padrão ocidental, composto por batata/aipim/inhame, macarrão, farinha/farofa/angu, pizza/hambúrguer/pastel, refrigerante/refresco e carne de porco/salsicha/linguiça/ovo, que explica 6,9% da variância; e (4) padrão lanche, composto por biscoito recheado, biscoitos tipo salgadinhos, chocolate e achocolatado, que explica 5,7% da variabilidade de consumo. O padrão alimentar lanche associou-se positivamente com o peso ao nascer (β = 56,64; p = 0,04) em gestantes adolescentes.

**CONCLUSÕES:**

Para as gestantes adolescentes, quanto maior a adesão ao padrão alimentar lanche durante a gestação, maior o peso ao nascer do bebê.

## INTRODUÇÃO

O peso ao nascer é importante indicador da sobrevivência infantil e reflete o risco de morte e agravos à saúde durante o primeiro ano de vida.^a^ Diversos fatores associam-se às variações no peso ao nascer, destacando-se o consumo alimentar materno.^15^ É essencial que a alimentação da gestante contenha energia e nutrientes suficientes para manter a saúde materna e permitir o crescimento e desenvolvimento fetal adequados.^7^ ^,^ ^30^ Avaliar o consumo alimentar, entretanto, é um desafio devido à complexidade da dieta humana, instigando pesquisadores a refinar os métodos de avaliação do consumo alimentar.

Historicamente, a maior parte dos estudos epidemiológicos tinha como foco principal avaliar a associação entre consumo de nutrientes e desenvolvimento de doenças.^20^ Contudo, essa avaliação é considerado reducionista, pois desconsidera a complexa interação entre nutrientes e alimentos. Isto ocorre porque indivíduos não ingerem isoladamente nutrientes e sim refeições compostas por grande variedade de alimentos que interagem entre si.^29^

Abordar o consumo alimentar através da identificação de padrões alimentares vem sendo descrito como método que mais se aproxima do complexo ato de se alimentar,^31^ visto que considera as diferentes combinações e composições das refeições. Além disso, o estudo de padrões alimentares permite a formulação de recomendações dietéticas baseadas em alimentos.^31^

Entretanto, poucos estudos têm avaliado o padrão de consumo alimentar entre as gestantes, particularmente suas repercussões sobre o feto.^10^ ^,^ ^22^

Este estudo objetivou analisar se padrões de consumo alimentar durante o terceiro trimestre gestacional estão associados ao peso ao nascer.

## MÉTODOS

Este estudo faz parte do projeto “Capital Social e Fatores Psicossociais associados à Prematuridade e ao Baixo Peso ao Nascer”, realizado nos municípios de Petrópolis e Queimados, RJ, entre dezembro de 2007 e agosto de 2008.^b^ Trata-se de uma coorte prospectiva com quatro ondas de seguimento: gestação, puerpério, três e seis meses pós-parto. Foram analisados dados da primeira (gestação) e da segunda etapa (pós-parto) de seguimento.

Foram convidadas a participar da coorte todas as gestantes com até 22 semanas de idade gestacional, atendidas nos serviços de pré-natal de 11 unidades de saúde da rede municipal do Sistema Único de Saúde (SUS), sendo oito em Queimados e três em Petrópolis, RJ, as quais detinham 90,0% da cobertura do cuidado pré-natal do SUS.

Para cálculo do tamanho amostral da coorte, considerou-se prevalência de 10,0% de nascimentos prematuros e com baixo peso, nível de significância de 5% e poder de 90,0%, para detectar diferenças de pelo menos 5,0%.^11^ Após ajustes para populações finitas e acréscimo de 20,0% para possíveis perdas durante o acompanhamento das gestantes, o tamanho amostral foi definido em 940 gestantes em Petrópolis e 840 em Queimados.

Devido a nascimentos prematuros (n = 167), perda de seguimento (n = 104), recusas (n = 71), abortos (n = 62), falta de informação do peso ao nascer (n = 31) e presença de gestação gemelar (n = 17), a amostra do presente estudo foi composta por 1.298 mulheres.

O consumo alimentar foi avaliado por meio de questionário de frequência de consumo alimentar (QFA) semiquantitativo, referente ao último trimestre gestacional, composto por 29 itens alimentares e obtido por simplificação do QFA de 80 itens, validado por Sichieri e Everhart.^28^ Para esta simplificação, foi feita análise secundária do banco de dados empregado na construção do QFA original, utilizando-se regressão *stepwise* que explicasse 95,0% da variância para os seguintes nutrientes: energia, carboidrato, lipídeos, ácidos graxos saturados, monoinsaturados e polinsaturados, colesterol, cálcio e vitamina C.^c^

A informação sobre o peso ao nascer foi extraída dos prontuários médicos. Quando não estava disponível, foram utilizados os dados da caderneta da criança ou da declaração de nascido vivo.

Foram avaliadas as seguintes variáveis de exposição: (i) sociodemográficas: idade das gestantes em anos, estado marital (vive com companheiro; não vive com companheiro), cor de pele autorreferida (branca; preta; parda), escolaridade em anos de estudo, trabalho fora de casa (não; sim), classe econômica (B ou C; D ou E); (ii) tabagismo (não; sim); (iii) doenças crônicas autorreferidas: hipertensão arterial (não; sim), diabetes mellitus (não; sim); (iv) antecedentes obstétricos: paridade (primípara; 2-3 filhos; 4 ou mais filhos), história anterior de óbito perinatal, baixo peso ao nascer ou prematuridade; (v) estado nutricional materno (IMC pré-gestacional e ganho de peso gestacional) e (vi) adequação do pré-natal. A história obstétrica e intercorrências na gestação, como diabetes e hipertensão arterial, foram referidas pela mulher na entrevista na maternidade após o parto, por meio da pergunta: “Você teve algum desses problemas de saúde na gravidez?” Pressão alta ou eclâmpsia (sim; não); Diabetes gestacional (sim; não).

A classificação socioeconômica seguiu critério adotado pela Associação Brasileira de Empresas de Pesquisa, baseado nos bens da família e no grau de instrução do seu respectivo chefe, constituindo-se escore final que define os grupos socioeconômicos A (o mais alto), B, C, D e E (o mais baixo).^1^ As classes socioeconômicas foram agregadas para análise multivariada em B ou C e D ou E, não tendo nenhuma mulher classificada na categoria A. Para avaliação da adequação do pré-natal foi utilizado o índice de Kotelchuck,^16^ que se baseia na idade gestacional no início do pré-natal e no percentual de adequação do número de consultas. Essas duas dimensões foram agrupadas em três categorias: pré-natal mais que adequado; adequado; e parcialmente inadequado e inadequado.

O dado de peso pré-gestacional foi obtido do cartão da gestante até a 13ª semana gestacional ou, quando indisponível, considerou-se o peso pré-gestacional autorrelatado. Esse peso foi utilizado para calcular o índice de massa corporal (IMC) prévio à gestação, permitindo classificar o estado nutricional da gestante em quatro categorias: baixo peso (IMC pré-gestacional < 18,5 kg/m^2^), peso adequado (IMC pré-gestacional entre 18,5 e 24,9 kg/m^2^), sobrepeso (IMC pré-gestacional entre 25 e 29,9 kg/m^2^) e obesidade (IMC pré-gestacional ≥ 30 kg/m^2^).^26^ A partir dessas informações, foram determinadas as faixas de adequação de peso para cada gestante, equivalente a ganho ponderal gestacional total de: 12,5 a 18 kg para mulheres com baixo peso pré-gestacional; 11,5 a 16 kg para mulheres eutróficas; 7 a 9 kg para mulheres com sobrepeso; e 7 kg para mulheres obesas.^26^ O peso ao final da gestação foi referido pela mulher durante a entrevista pós-parto. Para o cálculo do ganho de peso gestacional total, subtraiu-se o peso pré-gestacional do peso ao final da gestação.

Para a identificação dos padrões alimentares, os 29 itens listados no QFA foram agrupados em 20 grupos alimentares com base em suas características nutricionais e frequência de consumo.

Análise fatorial exploratória foi utilizada para derivar padrões alimentares baseados na porção diária de consumo de cada grupo alimentar. Uma matriz de correlação foi construída para verificar se as variáveis estavam correlacionadas e se o modelo fatorial era apropriado para os dados. Para isso, utilizou-se o teste de esfericidade de Bartlett (TEB) e a medida de adequação da amostra de Kaiser-Meyer-Olkin (KMO).

A extração dos fatores foi feita pela análise de componentes principais (ACP), ortogonalmente transformados, utilizando-se a rotação Varimax para obtenção de uma estrutura com independência dos fatores, facilitando a interpretação.

A definição dos fatores a serem retidos baseou-se no *scree plot* (gráfico que apresenta os autovalores em relação ao número de fatores em sua ordem de extração), no percentual da variância explicada pelos fatores e no conhecimento dos pesquisadores (considerações sobre interpretabilidade e simplicidade dos padrões). Realizou-se então nova análise fatorial fixando-se o número de fatores a serem extraídos.

Foram mantidos, nos fatores (padrões), os itens com carga fatorial superior a 0,30 e consideradas aceitáveis comunalidades mínimas de 0,25.^23^ A consistência interna de cada fator extraído foi verificada por meio do alpha de Cronbach. Posteriormente, os padrões foram nomeados com base na interpretação dos dados.

A associação entre padrões alimentares e peso ao nascer foi avaliada por modelo de regressão linear multivariado. Inicialmente, foi feita a análise de regressão linear bivariada entre todas as variáveis de exposição e o peso ao nascer, além de análise de regressão linear bivariada entre todas as covariáveis do estudo e os padrões alimentares com o intuito de detectar os possíveis fatores de confusão. Foi realizada análise distinta para cada padrão alimentar. Todas as covariáveis com p < 0,20 em ambas as análises foram consideradas potenciais candidatas para comporem o modelo final. O modelo final também foi mutuamente ajustado pelos demais padrões alimentares, dado que, nesse tipo de análise, os indivíduos participam de mais de um padrão e estes são não correlacionados, devido ao método de rotação empregado.

O estudo foi aprovado pelo Comitê de Ética em Pesquisa da Escola Nacional de Saúde Pública (ENSP/Fiocruz – CAAE 0156.0.031.000-06), estando de acordo com os princípios éticos contidos na Resolução 196/96, do Conselho Nacional de Saúde (CNS, 1996).

As análises estatísticas foram realizadas no programa estatístico *Statistical Package for Social Sciences* (SPSS) *forWindows*, versão 16.0.

## RESULTADOS

A idade média das mulheres entrevistadas foi 24,7 anos, a média de anos de estudo foi inferior a oito. A maioria das mulheres estava sem trabalho remunerado (59,7%), e pertencia às classes econômicas B ou C (66,4%). A maioria já possuía mais de um filho e 12,8% apresentava história anterior de recém-nascido com baixo peso ao nascer, prematuridade ou óbito perinatal (Tabela 1).

**Tabela 1:** Características sociodemográficas, antecedentes obstétricos, características da gestação e parto e do recém-nascido em coorte de gestantes. Petrópolis e Queimados, RJ, 2007-2008.

Variável	Total	
	n	%
Classe social		
C + B	862	66,4
E + D	436	33,6
Raça/Cor da pele		
Branca	440	33,9
Parda	557	42,9
Preta	301	23,2
Situação conjugal		
Vive com companheiro	957	73,7
Não vive com companheiro	341	26,3
Trabalho remunerado		
Não	775	59,7
Sim	523	40,3
História anterior de óbito perinatal, baixo peso ao nascer ou prematuridade*		
Não	635	87,2
Sim	93	12,8
Paridade		
Primípara	570	43,9
2 a 3	558	43,0
4 ou mais	170	13,1
Estado nutricional pré-gestacional		
Baixo peso	96	8,9
Eutrófica	768	71,2
Sobrepeso	214	19,9
Adequação do ganho de peso gestacional		
Insuficiente	358	32,5
Adequado	330	30,0
Excessivo	412	37,5
Adequação do pré-natal		
Mais que adequado	508	39,2
Adequado	436	33,7
Inadequado	351	27,1
Doença hipertensiva gestacional		
Não	1.051	81,0
Sim	246	19,0
Diabetes mellitus gestacional		
Não	1.266	97,5
Sim	32	2,5
Fumo na gestação		
Não	1.141	88,0
Sim	155	12,0
Tipo de parto		
Vaginal	723	55,7
Cesariana	575	44,3

	Média	DP

Idade (em anos)	24,7	6,1
Anos de escolaridade	7,8	2,9
Peso ao nascer (em gramas)	3,25	444,8

* Dados excluindo as primíparas.

Considerando-se a adequação do número de consultas de pré-natal para a idade gestacional (índice de Kotelchuck),^16^ mais de 70,0% das puérperas teve pré-natal adequado ou mais que adequado. Das entrevistadas, tabagismo foi relatado por 12,0% das mulheres (Tabela 1); 19,0% apresentaram diagnóstico de hipertensão arterial gestacional e 2,5%, de diabetes.

Observou-se que 28,8% das mulheres iniciaram a gestação com estado nutricional inadequado e, destas, 20,0% apresentavam sobrepeso. A média de ganho de peso na gestação foi de 13,3 kg, porém menos de 1/3 das mulheres apresentou ganho de peso considerado adequado e 37,5% apresentou ganho excessivo. Houve predomínio do parto vaginal (55,7%) e o peso médio ao nascer do recém-nascido foi 3.253 g (Tabela 1).

Foram identificados, inicialmente, sete fatores com autovalores maiores que 1,0 e que explicavam 51,3% da variância do consumo alimentar (dados não apresentados em tabela). Com base no *scree plot,* nos autovalores e considerando a interpretabilidade dos padrões, deveriam ser retidos quatro fatores que, juntos, explicavam 36,4% da variabilidade de consumo (Tabela 2).

**Tabela 2:** Matriz fatorial rotada, cargas fatoriais, variância e alpha de Cronbach para os quatro padrões alimentares identificados entre gestantes do município de Petrópolis e Queimados, RJ, 2007-2008.

Grupo alimentar	Carga fatorial			
	Prudente	Tradicional	Ocidental	Lanche
Leite	0,49			
Iogurte	0,62			
Queijo	0,59			
Biscoito sem recheio	0,48			
Carne de frango/Boi/Peixe/Fígado	0,42			
Fruta ou suco natural	0,56			
Feijão		0,51		
Arroz		0,61		
Açúcar		0,64		
Manteiga/Margarina		0,68		
Pão		0,58		
Vegetais		0,36		
Batata/Aipim/Inhame			0,60	
Macarrão			0,69	
Farinha/Farofa/Angu			0,55	
Refrigerante/Refresco			0,45	
Carne de porco/Salsicha/Linguiça/Ovo			0,44	
Pizza/Hambúrguer/Pastel			0,37	
Chocolate				0,61
Achocolatado				0,47
Biscoito recheado				0,68
Biscoito tipo salgadinho				0,65

Autovalores	3,28	1,94	1,53	1,26
% da variância explicada	14,94	8,80	6,94	5,74
% da variância acumulada explicada	14,94	23,74	30,68	36,42
Alpha de Cronbach	0,50	0,52	0,33	0,41

d World Health Organization. The global burden of disease: 2004 update. Geneva; 2008 [citado 2015 abr 17]. Disponível em: http://www.who.int/healthinfo/global_burden_disease/2004_report_update/en/

Os quatro padrões de consumo foram denominados e compostos da seguinte forma: (1) padrão prudente, composto por leite, iogurte, queijo, frutas e suco natural, biscoito sem recheio e carne de frango/boi/peixe/fígado, que explicava 14,9% do consumo; (2) padrão tradicional, composto por feijão, arroz, vegetais, pães, manteiga/margarina e açúcar, que respondeu por 8,8% da variação do consumo; (3) padrão ocidental, composto por pizza/hambúrguer/pastel, batata/aipim/inhame, refrigerante/refresco, macarrão, farinha/farofa/angu, carne de porco/salsicha/linguiça/ovo, responsável por 6,9% da explicação da variância; e (4) padrão lanche, composto por biscoito recheado, biscoitos tipo salgadinho, chocolate e achocolatado, explicando 5,7% da variância (Tabela 2).

Todos os grupos alimentares se associaram a algum dos quatro padrões identificados e apenas um item alimentar foi excluído da análise (café/chá preto ou chá mate) por apresentar comunalidade muito baixa, não se enquadrando em nenhum dos padrões definidos.

Na análise bivariada, considerando-se a significância estatística menor que 20,0%, o padrão prudente associou-se positivamente com classe econômica B ou C, trabalho remunerado, idade, escolaridade, pré-natal mais que adequado e excesso de ganho de peso na gestação, e associou-se inversamente com o fumo na gestação. As gestantes classificadas no padrão tradicional ou ocidental apresentaram algumas características comuns, como: maior paridade, fumo na gestação e menor obesidade pré-gestacional. Gestantes classificadas no padrão alimentar lanche eram significativamente mais jovens, com menos sobrepeso e obesidade pré-gestacional e mais pré-natal inadequado (Tabela 3).

**Tabela 3:** Estimativa dos parâmetros da regressão linear bivariada* entre padrão alimentar e variáveis maternas em coorte de gestantes. Petrópolis e Queimados, RJ, 2007-2008.

Variável	Padrão prudente		Padrão tradicional		Padrão ocidental		Padrão lanche	
	β	p	β	p	β	p	β	p
Classe social								
E + D	0,000		0,000		0,000		0,000	
C + B	0,309	< 0,001	-0,089	0,108	-0,171	0,002	0,084	0,177
Renda familiar *per capita*								
≤ 1/2 SM	-0,499	< 0,001	0,047	0,544	0,313	< 0,001	0,074	0,312
> 1/2 e ≤ 1 SM	-0,156	0,005	0,134	0,095	0,175	0,027	0,181	0,018
> 1 SM	0,000		0,000		0,000		0,000	
Raça								
Branca	0,000		0,000		0,000		0,000	
Parda	0,010	0,846	-0,035	0,507	0,049	0,355	-0,011	0,83
Preta	-0,126	0,039	-0,007	0,914	0,133	0,031	0,024	0,681
Idade (anos)	0,012	0,006	-0,006	0,143	-0,004	0,343	-0,047	< 0,001
Escolaridade (anos)	0,042	< 0,001	-0,003	0,723	-0,045	< 0,001	0,005	0,574
Situação conjugal								
Vive com companheiro	0,000		0,000		0,000		0,000	
Não vive com companheiro	-0,143	0,015	-0,008	0,891	0,025	0,676	0,191	0,001
Trabalho remunerado								
Não	0,000		0,000		0,000		0,000	
Sim	0,189	< 0,001	-0,016	0,758	-0,084	0,115	-0,157	0,002
Paridade								
Primípara	0,065	0,218	0,031	0,561	-0,080	0,128	-0,263	< 0,001
2 a 3	0,000		0,000		0,000		0,000	
4 ou mais	-0,199	0,008	0,093	0,218	0,314	< 0,001	0,302	< 0,001
Estado nutricional pré-gestacional								
Baixo peso	-0,005	0,960	0,037	0,713	-0,028	0,774	0,118	0,206
Eutrófica	0,000		0,000		0,000		0,000	
Sobrepeso	-0,123	0,092	-0,047	0,516	-0,062	0,394	-0,207	0,020
Obesa	-0,082	0,404	-0,217	0,027	-0,227	0,019	-0,305	0,000
Ganho de peso gestacional (kg)								
Insuficiente	-0,041	0,564	-0,026	0,722	0,000	0,996	-0,121	0,071
Adequado	0,000		0,000		0,000		0,000	
Excesso	0,094	0,177	0,067	0,342	0,046	0,509	-0,108	0,103
Adequação do pré-natal								
Mais que adequado	0,260	< 0,001	0,108	0,043	-0,194	< 0,001	-0,086	0,093
Adequado	0,000		0,000		0,000		0,000	
Inadequado	-0,270	< 0,001	-0,060	0,309	0,161	0,007	0,074	0,192
Doença hipertensiva na gestação								
Não	0,000		0,000		0,000		0,000	
Sim	-0,075	0,250	0,007	0,912	0,043	0,509	-0,073	0,247
Diabetes mellitus gestacional								
Não	0,000		0,000		0,000		0,000	
Sim	0,026	0,872	-0,547	< 0,001	-0,121	0,464	-0,248	0,116
História anterior de óbito perinatal, BPN ou PMT								
Não	0,000		0,000		0,000		0,000	
Sim	-0,029	0,255	-0,051	0,047	0,034	0,184	-0,053	0,038
Fumo								
Não	0,000		0,000		0,000		0,000	
Sim	-0,176	0,029	0,226	0,005	0,245	0,002	0,052	0,503
Tipo de parto								
Vaginal	0,000		0,000		0,000		0,000	
Cesariana	0,041	0,120	-0,082	0,02	-0,028	0,276	-0,060	0,022

β: coeficiente de regressão linear; BPN: baixo peso ao nascer; SM: salário mínimo; PMT: prematuridade

* Padrão alimentar como variável dependente e as demais variáveis como variáveis independentes.

Em relação ao peso ao nascer, mostraram associação positiva as variáveis classe social B ou C, idade materna, escolaridade, sobrepeso ou obesidade pré-gestacional, ganho ponderal excessivo na gestação e parto cesáreo. Contrariamente, não ter companheiro, ser primípara, iniciar o pré-natal com baixo peso, apresentar ganho de peso insuficiente, fazer pré-natal inadequado e fumar durante a gestação associaram-se negativamente com o peso ao nascer (Tabela 4).

**Tabela 4:** Estimativa dos parâmetros da regressão linear bivariada* entre peso ao nascer e variáveis maternas em coorte de gestantes. Petrópolis e Queimados, RJ, 2007-2008.

Variável	β	p
Classe social		
E + D	0,00	–
C + B	70,93	0,007
Renda familiar *per capita*	0,17	0,005
Raça		
Branca	0,00	–
Parda	-31,61	0,281
Preta	-14,44	0,563
Idade (anos)	5,00	0,014
Escolaridade (anos)	9,92	0,021
Situação conjugal		
Vive com companheiro	0,00	–
Não vive com companheiro	-76,48	0,006
Trabalho remunerado		
Não	0,00	–
Sim	4,22	0,867
Paridade		
Primípara	-58,76	0,018
2 a 3	0,00	–
4 ou mais	-11,34	0,758
Estado nutricional pré-gestacional		
Baixo peso	-46,93	0,318
Eutrófica	0,00	
Sobrepeso	122,40	< 0,001
Obesa	221,34	< 0,001
Ganho de peso gestacional		
Insuficiente	-77,41	< 0,001
Adequado	0,00	
Excesso	144,29	0,017
Adequação do pré-natal		
Mais que adequado	71,49	0,005
Adequado	0,00	–
Inadequado	-51,64	0,063
Doença hipertensiva na gestação		
Não	0,00	–
Sim	36,08	0,253
Diabetes mellitus gestacional		
Não	0,00	–
Sim	47,23	0,559
História anterior de óbito perinatal, BPN ou PMT		
Não	0,00	–
Sim	-67,52	0,159
Fumo		
Não	0,00	–
Sim	-133,71	< 0,001
Tipo de parto		
Vaginal	0,00	–
Cesariana	149,76	< 0,001
Sexo do bebê		
Masculino	0,00	–
Feminino	-111,39	< 0,001

β: coeficiente de regressão linear; BPN: baixo peso ao nascer; PMT: prematuridade

* Peso ao nascer como variável dependente e as demais variáveis como independentes.

As variáveis que apresentaram associação com significância de 20,0% tanto com o peso ao nascer quanto com os padrões alimentares e que entraram no ajuste do modelo final, além dos demais padrões alimentares, foram: classe econômica, idade, escolaridade materna, situação conjugal, paridade, estado nutricional pré-gestacional, adequação do pré-natal, histórico de óbito perinatal, baixo peso ao nascer ou prematuridade, fumo durante a gestação e tipo de parto (Tabelas 3 e 4).

Na análise multivariada, observou-se associação positiva entre consumo do padrão lanche e peso ao nascer, após ajuste por idade materna, escolaridade materna, situação conjugal, classe social, paridade, IMC pré-gestacional, adequação do pré-natal, história anterior de baixo peso/prematuridade, fumo durante a gestação, sexo do bebê e tipo de parto (dados não apresentados). Essa associação foi observada após a inclusão da idade materna no modelo, e não na análise bruta, o que mostra a presença de confusão negativa. Observou-se ainda que a distribuição da média do escore fatorial do padrão lanche também diferiu segundo faixa etária (0,44 nas mulheres entre 10-19 anos e -0,13 nas mulheres com 20 ou mais anos de idade) (dados não apresentados em tabela). Estes achados explicam a atenuação do efeito observada na análise bruta. Assim, o modelo final foi estratificado por idade materna (10 a 19 anos; 20 anos ou mais).

A análise de regressão linear multivariada ajustada para os fatores de confundimento mostrou associação positiva entre o padrão alimentar lanche e o peso ao nascer entre as gestantes adolescentes (Tabela 5).

**Tabela 5:** Modelo multivariado final entre peso ao nascer e padrão alimentar em coorte de gestantes. Petrópolis e Queimados, RJ, 2007-2008.

Padrão alimentar	Modelo bruto		Modelo ajustado* e estratificado por idade			
			10 a 19 anos		20 ou mais anos	

	β	p	β	p	β	p
Prudente	26,50	0,039	55,35	0,130	12,57	0,455
Tradicional	-9,23	0,462	11,45	0,723	19,90	0,241
Ocidentalizado	-15,12	0,214	15,88	0,623	10,17	0,554
Lanche	17,76	0,168	56,64	0,040	6,57	0,749

* Modelo multivariado ajustado para idade e escolaridade materna, situação conjugal, classe social, paridade, índice de massa corporal pré-gestacional, adequação do pré-natal, fumo durante a gestação, tipo de parto e padrões alimentares.

## DISCUSSÃO

Os resultados deste estudo mostram que, de forma geral, as gestantes atendidas nos serviços de pré-natal das unidades selecionadas dos municípios de Queimados e Petrópolis do Sistema Único de Saúde (SUS) eram jovens e de baixo nível socioeconômico, características semelhantes a de outros estudos realizados com gestantes e puérperas atendidas em unidades básicas do SUS.^17^ ^,^ ^24^

Com relação à avaliação antropométrica das mulheres, apesar de 64,9% delas terem iniciado a gestação com estado nutricional adequado, a prevalência de excesso de peso foi alta (26,9%). Esse resultado é semelhante ao encontrado no estudo de Nucci et al^21^ (2001) que, acompanhando 5.564 gestantes em seis capitais brasileiras, encontraram prevalência de 24,7% de sobrepeso e obesidade.

A média de ganho de peso gestacional (13,3 kg) assemelha-se à encontrada por Lacerda et al^17^ (2007) entre gestantes residentes na cidade do Rio de Janeiro, no ano de 2001 (13,4 kg). Os resultados referentes ao ganho de peso gestacional – 37,4% com ganho excessivo e 30,0% com ganho adequado segundo o IMC – estão de acordo com aqueles encontrados em outros estudos.^5^ ^,^ ^13^

As informações sobre o peso ao nascer mostram que, neste estudo, o peso médio (3.253 g) foi pouco superior (3.142 g) ao encontrado em outra localidade brasileira.^4^ Esta média de peso ao nascer mais elevada pode ser explicada pela exclusão de prematuros da amostra estudada. Ainda assim, o peso ao nascer médio encontrado neste estudo não atinge o intervalo de 3.400 a 3.500 g, valores esperados de peso ao nascer em condições ótimas de crescimento fetal.^d^

A análise fatorial permitiu identificar quatro padrões alimentares, que possuem alimentos em comum com padrões alimentares identificados em outras pesquisas.

O padrão prudente, também designado em outros estudos com gestantes como “saudável”, caracteriza-se pela presença de frutas, suco natural, vegetais, peixes e aves.^7^ ^,^ ^15^ ^,^ ^20^

O padrão tradicional, contendo feijão, arroz, vegetais, pães e manteiga/margarina, representa os alimentos que fazem parte do hábito alimentar brasileiro e se assemelha ao padrão tradicional, encontrado em estudos que utilizaram os mesmos procedimentos de análise para extração de padrões alimentares.^8^ ^,^ ^19^

O padrão ocidental é composto por alimentos com elevada densidade energética, baixo valor nutritivo (baixa quantidade ou isentos de micronutrientes) e de baixo custo financeiro, além de conter alimentos com elevada proporção de carboidrato (macarrão, batata/inhame/aipim, farinha/farofa/angu, refrigerante/refresco). Esse padrão também inclui alimentos fonte de proteínas, como carne de porco, ovo, linguiça e salsicha, sendo os dois últimos de baixa qualidade nutricional, pois contêm grande quantidade de gordura saturada, colesterol e sódio. Os alimentos *fast foods* (pizza/hambúrguer/pastel) também estão presentes nesse padrão e possuem baixo valor nutricional e elevada densidade energética.

O padrão lanche (biscoito recheado, biscoito tipo salgadinho, chocolate e achocolatado) também é composto por alimentos de baixo valor nutritivo e alta densidade energética. No estudo de Northstone et al,^20^ foi identificado o padrão alimentar denominado “confeitaria” (chocolate, doces, biscoitos e bolos) que possui alimentos em comum ao padrão lanche.

As variáveis selecionadas como fatores de confusão no modelo estatístico final apresentam um racional teórico. O peso ao nascer é um importante indicador de saúde, refletindo indiretamente as condições de vida das populações.^4^ Dessa forma, melhores situações socioeconômicas como maior nível de escolaridade materna, melhor classe social e maior acesso a serviços de saúde durante a gestação estão comumente associadas ao consumo alimentar saudável, bem como ao peso adequado ao nascer.^4^ ^,^ ^7^ ^,^ ^10^ ^,^ ^20^ A estrutura familiar também é um fator importante capaz de afetar tanto o consumo alimentar materno quanto o peso ao nascer. Mulheres que vivem com o companheiro e com menor paridade tendem a ter acesso a uma alimentação de melhor qualidade e a dar à luz a bebês com maior peso ao nascer.^6^ ^,^ ^10^ ^,^ ^20^ ^,^ ^27^ A idade materna precoce ou tardia bem como o tabagismo têm sido apontados como fatores de risco para desvios no peso ao nascer.^6^ Paralelamente, a literatura mostra que gestantes adolescentes, tabagistas e que consomem bebida alcoólica durante a gravidez têm pior padrão de consumo alimentar.^4^ ^,^ ^7^ ^,^ ^20^ O tipo de parto também influencia o peso ao nascer, pois em muitos casos a gestação é interrompida cirurgicamente antes do início do trabalho de parto, reduzindo o tempo de crescimento e ganho de peso intrauterino.^4^ Além disso, a cesariana é mais frequente entre as mulheres com intercorrências gestacionais como diabetes, hipertensão, anemia e outras complicações.^25^ Muitas dessas doenças são oriundas do consumo alimentar inadequado dessas mulheres.^3^ Por fim, o IMC materno também influencia diretamente tanto o consumo alimentar materno como o peso ao nascer.^4^ ^,^ ^9^

A análise da relação entre padrões de consumo alimentar e peso ao nascer mostrou que, para as gestantes adolescentes, quanto maior a adesão ao padrão lanche, maior o peso ao nascer do bebê. Possivelmente, esta associação foi observada apenas entre as gestantes mais jovens por serem estas as maiores consumidoras deste padrão, uma vez que apresentaram escore fatorial médio positivo, enquanto que esta média foi negativa dentre as mulheres com 20 ou mais anos de idade.

O padrão alimentar lanche é composto por alimentos com altas concentrações de carboidrato simples, lipídeos e baixas quantidades de proteína e micronutrientes. Essa combinação de nutrientes confere a esses alimentos uma elevada densidade energética, favorecendo o ganho de peso gestacional.

Uma dieta com grande quantidade de energia está associada a um maior ganho de peso gestacional^18^ que, por sua vez, está diretamente relacionado com o peso ao nascer do bebê.^26^ Isso ocorre porque o maior ganho de peso durante a gestação aumenta a disponibilidade e transferência de aminoácidos, glicose, ácidos graxos livres e triglicerídeos dos compartimentos maternos para os compartimentos fetais, aumentando seu crescimento e desenvolvimento.^14^ Segundo o IOM^26^ (2009), para cada aumento de 1 kg no GPG há um incremento de 16,7 a 22,6 g no peso ao nascer.

Uusitalo et al^32^ (2009) avaliaram a associação entre padrões de consumo alimentar e ganho de peso gestacional, identificando sete padrões, dos quais dois associaram-se ao ganho de peso gestacional: padrão *fast food* e padrão álcool e gordura. Os autores concluíram que as mulheres que pertenciam ao maior quartil de consumo do padrão *fast food* ganhavam em média 1,3 kg a mais do que as que pertenciam ao menor quartil deste padrão. Por outro lado, mulheres que pertenciam ao quartil de consumo mais elevado do padrão álcool e gordura ganhavam em média 0,7 kg a menos do que as que pertenciam ao menor quartil de consumo deste padrão.

Algumas limitações metodológicas deste estudo devem ser mencionadas. Dentre elas, o pequeno número de alimentos do QFA e a falta de porções padronizadas para todos os alimentos. Além disso, o QFA utilizado não foi testado quanto à acurácia na estimativa de alimentos, mas apenas de nutrientes. A despeito dessas limitações, os resultados obtidos pelo questionário são coerentes com o de outros estudos, como o realizado por Barros et al,^2^ que utilizaram questionário semelhante para avaliar o consumo alimentar gestacional de 1.228 adolescentes e também encontraram resultados coerentes com a literatura.

Outro fator importante que se relaciona à subjetividade, envolvendo a análise fatorial, é a especificidade dos resultados obtidos através desta técnica para a população investigada, dificultando a comparação entre os estudos. Além disso, apesar de as mulheres incluídas neste estudo viverem, majoritariamente, em situação de pobreza, os fatores referentes às características socioeconômicas e de estilo de vida apresentaram relação com hábitos alimentares.

Existe consenso na literatura de que a avaliação do consumo alimentar durante a gestação é importante e os estudos que abordam este tema ainda são escassos. Não foram encontrados estudos nacionais que avaliassem o efeito da alimentação materna sobre o peso ao nascer. Assim, esta pesquisa procurou contribuir com a determinação de padrões alimentares durante a gestação. A importância epidemiológica destes resultados, com base no estudo de coorte em dois municípios brasileiros, reside no fornecimento de subsídios para a racionalidade de estratégias nutricionais de intervenção mais apropriadas para gestantes.

## Footnotes

a World Health Organization.The global burden of disease: 2004 update. Geneva; 2008 [citado 2015 abr 17]. Disponível em: http://www.who.int/healthinfo/global_burden_disease/2004_report_update/en/

b Projeto: “Capital Social e Fatores Psicossociais Associados a Prematuridade e ao Baixo Peso ao Nascer”. Edital MCT/CNPq/MS-SDTIE-DECIT 26/2006 – Estudo de Determinantes Sociais da Saúde, Saúde da Pessoa com Deficiência, Saúde da População Negra, Saúde da População Masculina. Processo nº 409805/2008-8.

c Consultoria fornecida por Sichieri R para desenvolvimento do questionário simplificado de consumo alimentar, em 1998.
